# Challenges related to resources mobilization for measles outbreak response: Madagascar experience during the 2018-2019 measles outbreak

**DOI:** 10.11604/pamj.2020.36.304.24514

**Published:** 2020-08-19

**Authors:** Vincent Dossou Sodjinou, Marcellin Mengouo Nimpa, Alfred Douba, Yolande Vuo Masembe, Mireille Randria, Charlotte Faty Ndiaye

**Affiliations:** 1 World Health Organization Regional Office for Africa, Brazzaville, Congo,; 2 World Health Organization Country Office, Madagascar,; 3 Felix Houphouet Boigny University, Abidjan, Côte d´Ivoire

**Keywords:** Measles, outbreak, resources mobilization, Madagascar

## Abstract

**Introduction:**

on October 4^th^, 2018, a measles outbreak was declared in Madagascar. This study describes challenges related to resources mobilization for the outbreak response.

**Methods:**

data were collected using minutes of coordination committee meetings, activities reports, operational action plans and situation reports.

**Results:**

the total cost of the outbreak response was estimated to US$ 11,281,381. Operational cost was the leading cost driver (42.45%) followed by vaccine cost (33.74%). Cases management, epidemiological surveillance, communication and social mobilization and routine immunization strengthening represented 23.81% of the total cost. The main funder of the outbreak response was the measles and rubella initiative.

**Conclusion:**

good coordination, open dialogue, good use of financial resources and accountability of government and partners have enabled to gain the confidence of national and international donors.

## Introduction

Measles is one of the most infectious human diseases and can cause serious illness, lifelong complications and death [1]. Previous to the availability of measles vaccine in 1963 [2,3], measles infected over 90% of children before they reached 15 years of age and cause more than two million deaths and between 15,000 and 60,000 cases of blindness annually worldwide [1,4]. In 2000, measles-related deaths were estimated to 535,000 by the World Health Organization (WHO). The majority of these deaths occurred in developing countries and this burden accounted for 5% of all under five mortality [1,5]. Recognizing that deaths and disabilities caused by measles and rubella are completely preventable with safe and inexpensive vaccines, the measles and rubella initiative (formerly, the measles initiative) was launched in 2001 to support technically and financially accelerated measles control activities [6,7]. The 2012-2020 strategic plan explains how countries, working together with the Measles and Rubella Initiative (MRI) and its partners, will achieve a world without measles, rubella and congenital rubella syndrome. The strategy focuses on the implementation of five core components: achieve and maintain high levels of population immunity by providing high vaccination coverage with two doses of measles- and rubella-containing vaccines; monitor disease using effective surveillance and evaluate programmatic efforts to ensure progress; develop and maintain outbreak preparedness, respond rapidly to outbreaks and manage cases; communicate and engage to build public confidence and demand for immunization; perform the research and development needed to support cost-effective operations and improve vaccination and diagnostic tools [1].

As a result of the MRI and its partners´ effort, measles deaths dropped by 84 percent worldwide, from 550,100 deaths in 2000 to 89,780 in 2016 [8]. Despite these remarkable achievements, measles remains a serious public health threat globally, since measles outbreak occurred over the five past years in many developing countries and developed countries as well [9,10]. According to the WHO, over the first quarter of 2019, more than 110,000 measles cases were reported worldwide, representing about 300 percent increase from the same period in 2018 [11]. Within this global context of increase number of measles cases, an unprecedented measles outbreak occurred in October 2018 in Madagascar who has been implementing measles rubella elimination strategies. The previous measles outbreak occurred in the country in 2003 [12]. The outbreak affected all the 22 regions and 97% (111 districts out of 114) of health districts. As per Madagascar Ministry of Health and WHO report, 135,067 cases of measles and 884 deaths have been reported between 3^rd^ September 2018 and 14^th^ April 2019 and children aged 1 to 4 years most affected [13]. This study describes the Madagascar experience in resource mobilization to support measles outbreak response activities and best practices and lessons learned which could be helpful for other countries.

## Methods

**Setting:** Madagascar is the fourth biggest island country in the world. Located in the Indian Ocean, it covers 587041km^2^ and is separated from the African continent by the Mozambique channel. The country is divided in to 22 administrative regions and 114 health districts. In 2018, the total population was estimated at 26,330,637 inhabitants with 49.9% males and 50.1% females. Twenty percent (20%) of the population live in urban area and 80% reside rural area [4].

**Data collection:** data were collected using minutes of coordination committee meetings, activities reports, operational action plans and situation reports.

**Outbreak management:** outbreak confirmation was followed by a rapid grading of the outbreak within 72 hours by the WHO which classified it as a grade two outbreak, meaning that country need support to cope with this outbreak. Therefore, outbreak management by the ministry of health and partners was based on the WHO Incidence Management system.

**Coordination and resources mobilisation:** the government and partners set up a three level coordination mechanism with task forces: a strategic coordination committee, a technical coordination committee and multisectoral coordination committee. The first two task forces, under the leadership of the ministry of health included the ministry of health and all the partners including the private sector, had weekly meeting. The multisectoral committee was under the leadership of the prime minister and included different departments and bilateral and multilateral partners. Beside theses task forces, WHO established weekly partners´ coordination meeting to ensure coordinated support to the government and complementarity of partners interventions.

## Results

**Figure 1:**
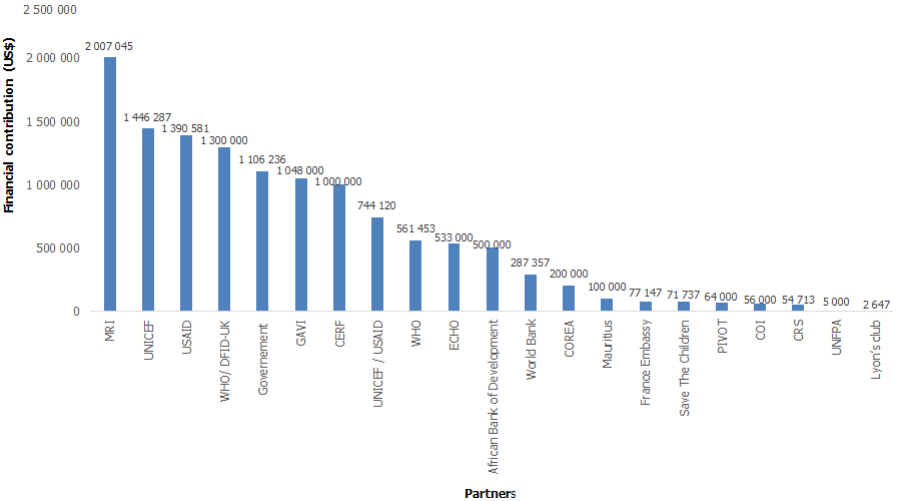
distribution of financial contribution by partners and government during measles outbreak, Madagascar, 3^rd^ September 2018 to 30^th^ May 2019

The total cost of the outbreak response was initially estimated to US$11,281,381 (Table 1). Operational cost was the leading cost driver (42.45%) followed by vaccine cost (33.74%). Cases management, epidemiological surveillance, communication and social mobilization and routine immunization strengthening represented 23.81% of the total cost. Measles outbreak response activities required both local and external financial resources with 91.19% from partners and 8.81% from government. Strong collaborative work of all involved stakeholders under the lead of the MoH led to a coordinated mapping of partners and interventions, joint resources mobilization, efficient running on the emergency operation centre and regular national committee coordination meeting for decision making. This strengthened coordination led to an effective team spirit between MoH and partners and enabled the effective implementation of response interventions. A total of US$ 12,555,323 were finally mobilized for the management of measles outbreak in Madagascar (an initial total cost increase of 11.29% used for routine immunization strengthening). This amount come from 21 partners and the government of Madagascar. Financial contribution ranged from a minimum of US$ 2,647 to a maximum of US$ 2,007,045. The four main contributors were MRI (16.00%), UNICEF (11.52%), USAID (11.08%) and WHO/DFID-UK (10.35%) (Figure 1).

**Table 1:** distribution of initial financial resources of the outbreak response by item, Madagascar, 2018-2019

Item	Vaccine cost (US$)	Operational cost (US$)	Total cost (US$)
Vaccination campaign round 1	1,077,079	1,278,910	2,355,989
Vaccination campaign round 2	671,206	835,333	1,506,539
Vaccination campaign round 3	2,057,574	2,674,651	4,732,225
Cases management	-	-	835,367
Epidemiological surveillance	-	-	192,266
Communication and Social mobilization	-	-	388,082
Routine immunization strengthening	-	-	1,270,913
Total cost (US$)	3,805,859	4,788,894	11,281,381

In Madagascar, the leading cost of the 2018-2019 measles outbreak response was the operational cost

## Discussion

Madagascar government made significant contribution to the measles outbreak response. This contribution represent 8.81% (US$ 1,106,236) of the total financial resources required for the outbreak response. This unexpected expense could have an impact in terms of cancelation or delay in the implementation of some health activities. In fact, every year, as in other African countries, the health sector is allocated a budget for the implementation of activities which are already identified. Thus, the budget can be broken down into operating and investment budget. Therefore, in the occurrence of an emergency such as an epidemic, if the country does not have a specific budget to deal with unexpected emergency, a proportion of the operating or investment budget has to be redirected toward the unexpected emergency. Consequently, some planned activities could be delayed or canceled. The financial contribution of the government to the measles outbreak could be a financial constraint in a context of scarcity of resources since Madagascar ministry of health faces a steady decrease of its budget since 2015 [14]. The financial constraint faced by the government of Madagascar during the outbreak was expressed by the secretary general of the ministry of health, at a national coordinating committee in March 2019, in terms of financial gap of more than US$ 1.5 million and requested the contribution of all partners to participate in mobilizing resources for social mobilization and vaccination mas campaign [15]. The MRI provided the largest financial contribution among partners. In fact, the MRI can fund a maximum of 50% of the generally accepted range of operational costs per targeted person during a measles outbreak when the country expresses a need [16].

WHO leadership and partner´s coordination role enhanced government and partners´ capacity to mobilize funds through effective information management and planning. WHO organized key advocacy interventions towards different donors including DFID, AFDB, Korean embassy, Japan embassy and Turkish embassy to improve donor´s awareness and commitment to fund the response. Information letters were developed and shared with key partners and audience were requested for further exchanges. These actions let to mobilization of resources from DFID, Korean embassy, AFDB and CERF. The organization internal funds were also mobilized. This incredible role of WHO in resource mobilization was reported in other emergencies. In Nigeria, WHO played a major role in resource mobilization for the polio eradication initiative over the period 2008-2015 [17]. The lead of the WHO in partners´ coordination has been done in accordance to one of its 6 core functions which is to providing leadership on matters critical to health and engaging in partnerships where joint action is needed [18]. In Madagascar, the WHO country office applied a dialogue approach with partner and government to obtain their engagement and effective financial contribution. This dialogue approach was applied by the WHO regional office to get support from a wide variety of partners to deal with Ebola virus disease outbreak which occurred in West Africa in 2014 [18]. In Nigeria, to manage the risks of donor fatigue, the WHO used the support of major donors in advocating with high-level government officials and high-profile donors to reinvigorate their financial commitment to the polio eradication initiative [17]. This strategy has also been applied by the WHO country office of Madagascar during the measles outbreak. Coordination which has been identified as a best practice from the polio eradication initiative [19] played a major role in financial resources mobilization during measles outbreak response in Madagascar.

Positioning measles outbreak as a global priority was challenging in a country who did not have any for 15 years. The 2018-2019 measles outbreak suggests that Madagascar was experiencing a quiet period and the low immunity levels from inadequate vaccination coverage resulted in a large proportion of the population susceptible to measles infection [20]. The outbreak was qualified grade 2 by the WHO. This grade means that the outbreak had moderate public health consequences that requires a moderate WHO country office response and/or moderate international WHO response [21]. The financial support related to this grade was the access to regional WHO financial resources and international resources mobilization on request [21]. The most obvious financial partner during this outbreak was the MRI. According to the MRI outbreak response fund standard operating procedure, “all GAVI eligible countries that have a significant measles outbreak of national public health importance and cannot respond to the outbreak fast enough with in-country funding (domestic epidemic response funds or donor funding) are eligible to request funding for outbreak response” [16]. GAVI eligible countries should also consider using health system and immunization strengthening funds for outbreak response activities especially outbreak investigation [22].

There were financial gaps at some points during the outbreak response [15]. Judicious and transparent use of resources and accountability of government and partners has enabled to progressively gain the confidence of national and international donors. To ensure the proper use of financial resources, the MRI specified in its standard operating procedure for outbreak response fund that the management and decision making regarding the allocation of the outbreak response funds are under the responsibility of the MRI core funding partners including the WHO [16]. Adequate financial resources are needed for adequate outbreak response but permanent fund is necessary for a disease control. For instance, in the United States of America, a periodic funding and disease cycle occurred before funding was held steady and reliably available for adequate preventive vaccination. In this cycle, measles outbreaks led to increased funding for measles control which then resulted in decreased measles cases and a perception by policy makers that funding could be reduced. This decrease in funding in turn led to a build-up of individuals susceptible to measles and another large outbreak [22]. Therefore, countries and partners should create a specific fund for measles control and not only for measles outbreak response in order to achieve the goal of measles elimination in each WHO region.

**Strengths and weaknesses of the study:** strengths of our study stem from the source of information. Data used for this study are those officially reported by Madagascar government and partners. Results of our study should be used taking into account its limitations. The total cost of the measles outbreak response may have been underestimated or over estimated. Since information sources were reports and documents related to the outbreak and not the payment receipts we were able to compute the exact amount of expenses. The study did not include expenses covered by the WHO regional office and related to international consultants. The total cost did not also include families´ expenses (treatment and burial).

## Conclusion

The measles response required joint financial resources from both Madagascar government and partners. The main funder of the outbreak response was the Measles and Rubella Initiative. Among partner, the coordination was led by the WHO. Good coordination, open dialogue, good use of financial resources and accountability of government and partners have enabled to gain the confidence of national and international donors.

### What is known about this topic

- A country with a grade two outbreak needs external support (human, material and financial);
- Resources mobilization during outbreak is challenging;
- Resources are provided by both government and partners.

### What this study adds

- Good coordination, open dialogue and good use of financial resources have enabled to gain the confidence of national and international donors;
- An estimated cost of a country-wide outbreak in a low income country of about 26 million inhabitants.
